# Telemedicine During the COVID-19 Pandemic: Impact on Care for Rare Cancers

**DOI:** 10.1200/GO.20.00220

**Published:** 2020-07-08

**Authors:** Alannah Smrke, Eugenie Younger, Roger Wilson, Olga Husson, Sheima Farag, Eve Merry, Aislinn Macklin-Doherty, Elena Cojocaru, Amani Arthur, Charlotte Benson, Aisha B. Miah, Shane Zaidi, Spyridon Gennatas, Robin L. Jones

**Affiliations:** ^1^ The Royal Marsden Hospital NHS Trust, London, United Kingdom; ^2^ Sarcoma Patients Euronet e.V./Association, Wölfersheim, Germany; ^3^ Institute of Cancer Research, London, United Kingdom

## Abstract

**PURPOSE:**

Many patients with cancer, often those with rare cancers such as sarcomas, travel long distances to access expert care. The COVID-19 pandemic necessitated widespread changes in delivery of cancer care, including rapid adoption of telemedicine-based care. We aimed to evaluate the impact of telemedicine on patients, clinicians, and care delivery at the Royal Marsden Hospital (RMH) Sarcoma Unit during the pandemic.

**METHODS:**

Data were extracted from patient records for all planned outpatient appointments at the RMH Sarcoma Unit from March 23 to April 24, 2020. Patients and clinicians completed separate questionnaires to understand their experiences.

**RESULTS:**

Of 379 planned face-to-face appointments, 283 (75%) were converted to telemedicine. Face-to-face appointments remained for patients who needed urgent start of therapy or performance status assessment. Patients lived on average > 1.5 hours from RMH. Patient satisfaction (n = 108) with telemedicine was high (mean, 9/10), and only 48% (n = 52/108) would not want to hear bad news using telemedicine. Clinicians found telemedicine efficient, with no associated increased workload, compared with face-to-face appointments. Clinicians indicated lack of physical examination did not often affect care provision when using telemedicine. Most clinicians (n = 17; 94%) believed telemedicine use was practice changing; congruently, 80% (n = 86/108) of patients desired some telemedicine as part of their future care, citing reduced cost and travel time.

**CONCLUSION:**

Telemedicine can revolutionize delivery of cancer care, particularly for patients with rare cancers who often live far away from expert centers. Our study demonstrates important patient and clinician benefits; assessment of longer-term impact on patient outcomes and health care systems is needed.

## INTRODUCTION

The development of telemedicine, defined as delivery of health services using communication technology,^1^ has overcome geographic and socioeconomic distances to improve access to care. During the SARS-CoV-2 pandemic, physical distancing^2^ was recommended to reduce the spread of coronavirus disease (COVID-19).^3^ Early evidence suggested patients with cancer were at higher risk for severe COVID-19.^4^ This resulted in rapid conversion of many outpatient oncology appointments to telemedicine.^5^ However, a paucity of literature exists related to telemedicine in oncology,^6^ particularly for management of the approximately 25% of patients with rare cancers^7,8^ and those who travel long distances to access care.

**CONTEXT**

**Key Objective**
What is the impact of rapid enforced telemedicine use during the COVID-19 pandemic on patients, clinicians, and health systems?
**Knowledge Generated**
In this retrospective case series of patients with sarcoma, the majority of patients were reviewed using telemedicine. Clinicians indicated telemedicine appointments were generally shorter than face to face, did not increase their workload, and lack of physical examination did not affect patient care. Both patients and clinicians reported satisfaction with telemedicine-based care and wished for telemedicine to be incorporated into future practice.
**Relevance**
These results necessitate a change of practice for future oncology care delivery to include telemedicine-based care, particularly for patients with rare cancers such as sarcomas.

Patients with rare cancers often require management at expert centers.^9^ However, because patients often live a long distance from such centers,^10,11^ geography can be a barrier to care. Sarcomas are a distinct group of rare cancers with > 50 histologic subtypes.^12^ The Royal Marsden Hospital (RMH) Sarcoma Unit treats patients from throughout the United Kingdom. Before the pandemic, telemedicine-based care in medical and radiation oncology clinics was not routine. The aim of this study was to review the adoption of telemedicine in the RMH Sarcoma Unit during the COVID-19 pandemic to provide an understanding of patient, provider, and health system experience.

## METHODS

Institutional ethical approval was obtained before study commencement (SE939/SE940). The decision to convert to a telemedicine appointment was at the discretion of the treating physician in line with National Institute for Health and Care Excellence Guidance^5^ and local institutional policy. Patients were offered a telemedicine appointment in advance of their regularly scheduled appointment. Generally, appointments for patients requiring urgent assessment or for those with significant radiologic or clinical progressive disease remained face to face. Data were extracted from the electronic patient record for all planned appointments (medical and radiation oncology) for patients age > 18 years between March 23 (commencement of UK lockdown) and April 24, 2020. Average travel times and distance from patient address (first 3 digits of postcode) to RMH were calculated using Google Maps.

The proposed survey was tested with patients and modifications were made. Patients with a clinic appointment were invited by phone over a 2-week period to consent to participate in an anonymous patient experience survey (online or paper), including a telemedicine section (Data Supplement). Clinicians in the sarcoma unit were provided an anonymous electronic survey via e-mail (Data Supplement). Data cutoff date for survey participation was May 1, 2020. Descriptive statistics were used.

## RESULTS

### Telemedicine Use in Sarcoma Clinics

A total of 316 patients had 379 appointments, with a median age of 59 (range, 19-92) years and slightly more females (n = 214/379; 56%; Table 1). Most patients had advanced disease (n = 258/379; 68%). Average travel time by car was 108 minutes. Telemedicine (phone) was used for 283 (75%) of appointments; most patients were under active surveillance (n = 131/283; 46%) or on oral therapy (n = 79/283; 28%; Table 1). Few patients (n = 3/286; 1%) declined telemedicine appointments (2 active surveillance and 1 oral therapy). A minority of telemedicine appointments (n = 13/283; 5%) were performed for results of progressive disease. Half of telemedicine appointments (n = 145/283; 51%) were performed by a clinician who had never met the patient. Face-to-face appointments were most commonly due to patients receiving intravenous systemic therapy (n = 30/96; 31%), results indicating disease progression (n = 29/96; 30%), or assessment of performance status (n = 15/96; 16%).

**TABLE 1:**
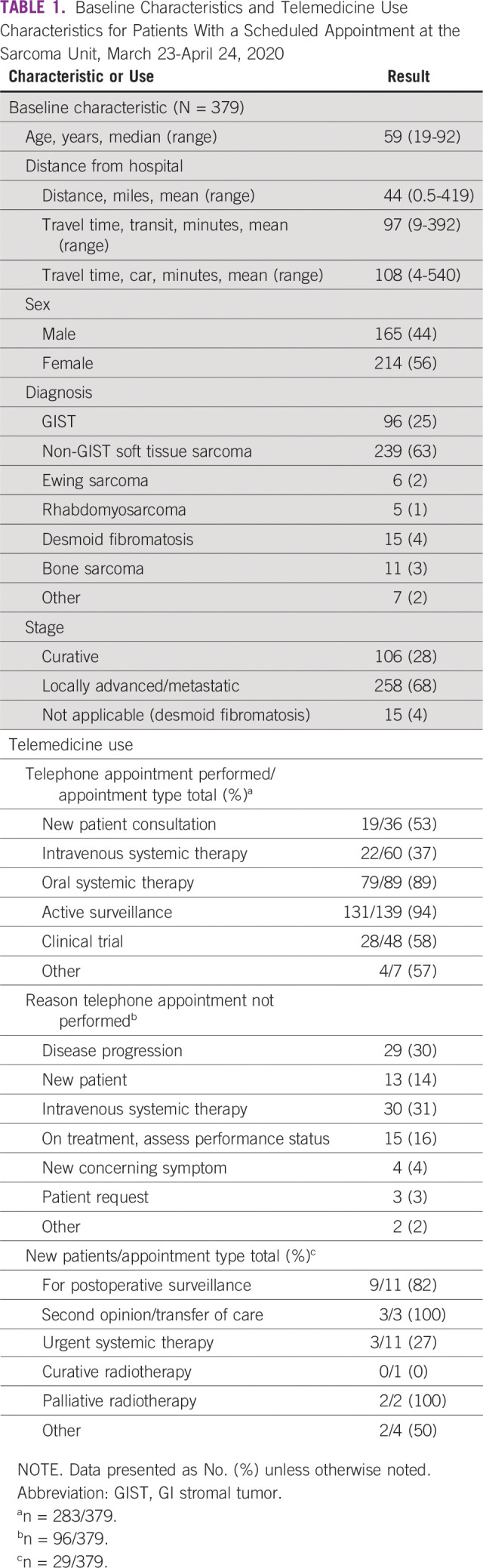
Baseline Characteristics and Telemedicine Use Characteristics for Patients With a Scheduled Appointment at the Sarcoma Unit, March 23-April 24, 2020

### Patient Survey on Telemedicine Experience

**FIG 1:**
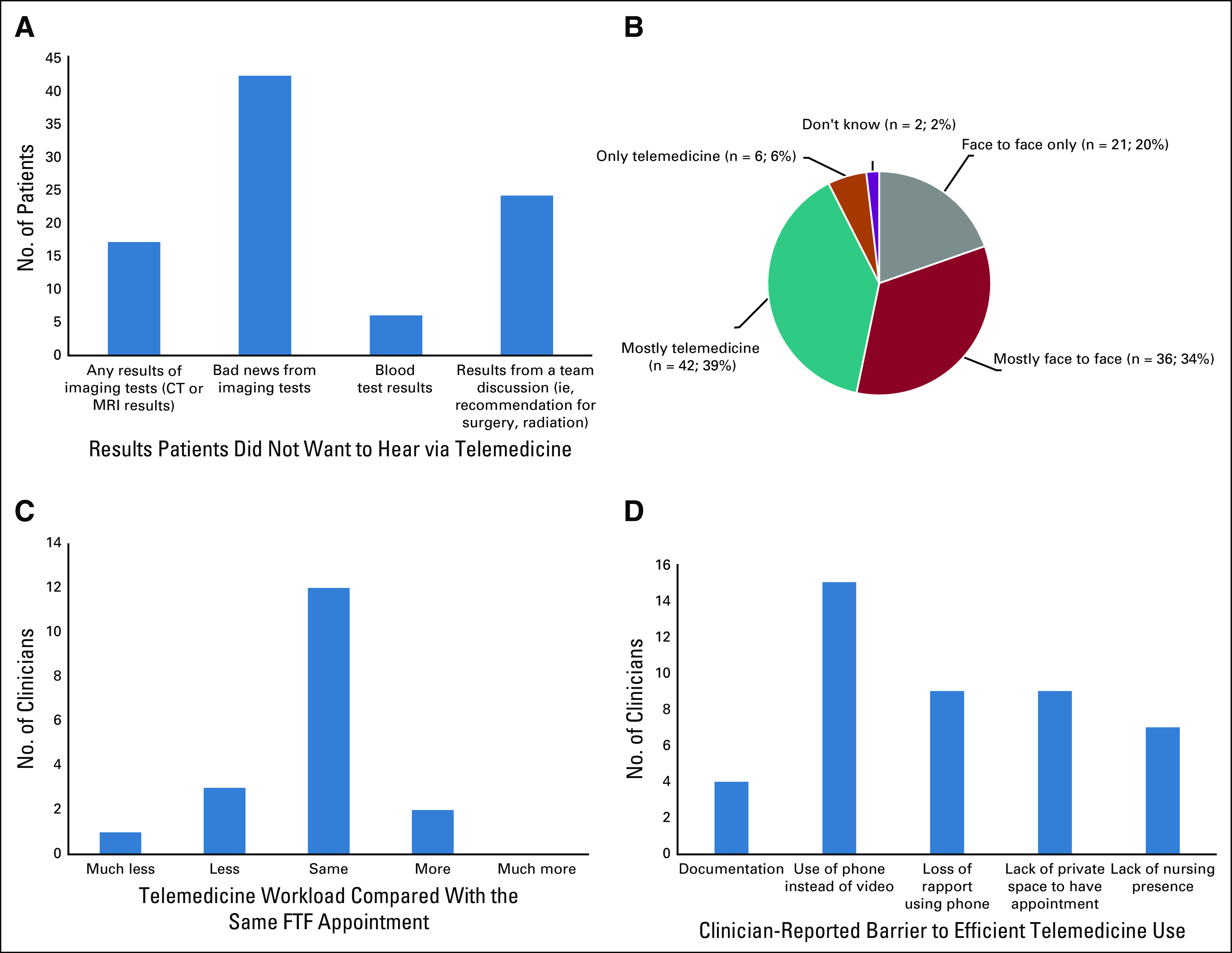
Key patient and provider survey results that inform current and future use of telemedicine-based care for patients with sarcoma and other rare cancers. (A) Types of information patients would not wish to hear using telemedicine. (B) Patient preference for future appointments. (C) Change in clinician-reported workload for telemedicine versus face to face (FTF). (D) Clinician-reported barriers to telemedicine-based care. CT, computed tomography; MRI, magnetic resonance imaging.

A total of 270 patients were invited, and 248 patients gave verbal consent to receive the survey. Response rate was high for electronic invitations (105/215; 49%) and lower for paper (3/33; 9%) at the cutoff date May 1, 2020. One hundred eight patients with a median age of 59 (range, 19-86) years participated (Table 2). Telemedicine appointments (n = 70) were more common than face-to-face appointments (n = 34). Mean satisfaction with telephone consultation was higher than face-to-face consultation (rating 8.99/10 *v* 8.35/10, respectively). The majority of patients (n = 86; 80%) indicated that they would like at least some future appointments to be performed using telemedicine (Fig 1B). Common reasons for telemedicine preference were reduced travel time (n = 45; 42%), reduced travel expenses (n = 21; 20%) and convenience (n = 32; 30%). Patients who preferred face-to-face appointments felt that face to face would be more reassuring (n = 45; 42%) and their treatment plan would be clearer (n = 21; 20%). Although sex or education level did not affect choice for method of consultation, patients who preferred face to face only were slightly older (median age, 69 years) than those who preferred at least some telemedicine (median age, 58 years). Almost half (n = 42; 48%) would not want to hear bad news over the phone, with no difference based on age, sex, or education level. Few patients (n = 17; 20%) would not want to hear any scan results (Fig 1A).

**TABLE 2:**
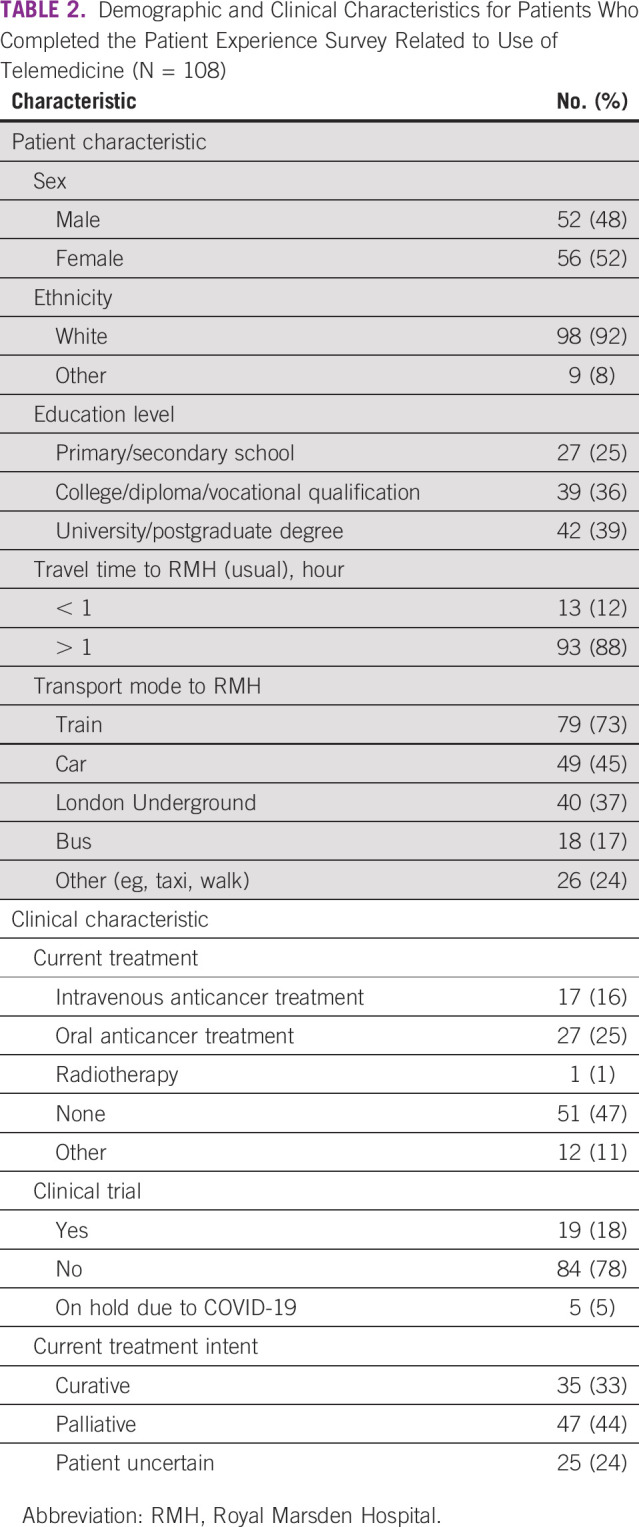
Demographic and Clinical Characteristics for Patients Who Completed the Patient Experience Survey Related to Use of Telemedicine (N = 108)

### Provider Survey on Telemedicine Experience

All invited clinicians (18/18; 100% response rate) participated in the survey, most were physicians (4 consultants, 4 clinical research fellows, 4 residents), and the remainder were nurses (2 nurse specialists, 4 research nurses); most had worked on the unit for > 5 years (n = 7; 39%) or < 2 years (n = 8; 44%). Overall, 78% (n = 14) found telemedicine appointments shorter than comparable face-to-face visits, with no difference on the basis of role or length of time working on the unit. Overall, 89% (n = 16) felt that telemedicine did not increase workload (Fig 1C), and, specifically, the majority of physicians (n = 10/12; 83%) indicated workload was the same as face to face. There was no difference in reported change in workload on the basis of length of time working on the unit. Clinicians reported that the inability to perform physical examination only rarely (n = 7; 39%) or sometimes (n = 8; 44%) affected telemedicine-based appointments for this cohort of patients (Fig 1D). Although there was no difference in response on the basis of clinician role, those who had worked on the unit for > 5 years were more likely to report physical examination rarely affected patient care than those who had worked for < 5 years (n = 4/7 [57%] *v* n = 2/8 [25%] of clinicians, respectively). Most clinicians indicated lack of video-based assessment was a barrier to care (n = 15; 83%; Fig 1D). The majority (n = 17; 94%) indicated telemedicine should become part of regular practice, with most favoring its use for follow-up of patients on active surveillance (n = 16; 89%) or stable doses on oral anticancer medication (n = 16; 89%). More than one-third (n = 7; 39%) desired nurse presence with patient for all telemedicine appointments. Most desired a separate assigned telemedicine clinic (n = 12; 67%) rather than appointments within existing face-to-face clinics (n = 6; 33%).

## DISCUSSION

Although this study was performed to assess an enforced change to practice due to the COVID-19 pandemic, our data highlight encouraging patient and provider satisfaction with telemedicine-based care. Clinician workload was not increased, with telemedicine appointments shorter than equivalent face-to-face appointments. Patients indicated similar satisfaction with face-to-face and telemedicine-based appointments. Congruency was seen between extracted and survey-obtained patient demographics, demonstrating that survey participants were representative of patients with appointments during the pandemic. Multiple types of appointments were held using telemedicine, including new patient consultations and those revealing progressive disease. Although an important limitation of telemedicine is lack of physical examination, clinicians generally did not believe that this affected delivery of care for the patients selected for telemedicine consultations. Face-to-face appointments appropriately continued for certain patients who required physical examination or assessment of performance status.

Beyond the COVID-19 pandemic, telemedicine-based care should be considered for certain patients, such as those receiving long-term oral therapies or active surveillance. The majority of patients indicated preferences for a combination of telemedicine and face-to-face appointments, and it is interesting that more than half of patients did not decline hearing negative results on the phone. This finding in particular is against many of the dogmatic principles of “breaking bad news”^13^ and is a key observation for understanding patients appropriate for telemedicine-based care.

Integration of telemedicine into routine care requires careful planning. Clinicians indicated that specialized nursing presence during telemedicine-based care was important. Patients with rare cancers have reported specialized nursing is supportive and provides specialized knowledge that may be lacking in their own local general practitioners.^14^ Nursing presence is also helpful for management of adverse effects of anticancer treatment, such as hand-foot syndrome for patients receiving long-term oral tyrosine kinase inhibitors. Need for nurse presence could be identified by clinicians or patients before the appointment, for example during “bad news” appointments, the first visit after starting a new systemic therapy, or a new patient consultation. Clinicians indicated a strong preference for video-based appointments instead of telephone, with the majority citing infrastructure (ie, physical quiet space, headset) as a major barrier to integration into practice. Video-based telemedicine may require information technology upgrades across health systems; however, multiple video-call–based platforms exist, and we expect these to continue to be adopted widely.^6^ For patients on stable doses of medications, pharmacy involvement will be critical to ensure patient access to a consistent supply. Furthermore, the impact of converting face-to-face to telemedicine-based assessments on provider remuneration must be considered.

Our experience with telemedicine-based care provision in sarcomas can serve as a model for other rare cancers. Similar to other patients with rare cancers,^11^ patients treated by our unit travel long distances for care. Patients preferred telemedicine because of travel distance and cost. Telemedicine use for 1 month during the pandemic saved, on estimate, 915 travel hours for 283 patients. Broadly, integration of a component of telemedicine to standard practice may have cost-saving implications to health care systems and reduce the carbon footprint, while increasing access to care for patients who live distant from their treating center. Although telemedicine-based care can improve access to care on the individual level, we acknowledge that access to care for patients with rare cancers is not equal across countries. Learning from the experience of telemedicine during the pandemic should be a reminder to clinicians that telemedicine-based networks for providers in countries with few designated rare cancer clinics may also be feasible to improve access to specialist knowledge.

Our study provides unique insight into the effect of enforced telemedicine-based care during the pandemic. This experience was generally positive for patients and clinicians. Integration of telemedicine-based care into daily practice may have particular importance for patients with rare cancers and those who live distant from care centers. Enforced adoption of telemedicine has demonstrated the time- and cost-saving implications for patients, which have the potential to revolutionize cancer care delivery.
